# Scrotal Dartos-Fascio-Myo-Cutaneous flaps for penis reconstruction after iatrogenic skin shaft sub-amputation

**DOI:** 10.1093/jscr/rjz206

**Published:** 2019-07-23

**Authors:** Mohamad Moussa, Mohamed Abou Chakra

**Affiliations:** 1 Department of Urology, Zahra University Hospital, Beirut, Lebanon; 2 Department of Urology, Faculty of Medical Sciences, Lebanese University, Beirut, Lebanon

## Abstract

Penile amputation is an uncommon genital injury, the causes of penile trauma are varied; it can be iatrogenic or caused by traffic accidents, burns, circumcision, animal bites, gunshots or self‐mutilation. The type and extent of penile trauma vary from mild to severe injuries, sometimes even with total amputation. A wide variety of surgical options exist for penile reconstruction. Often, not only the surgical but also psychological aspects of treatment will determine the success or failure of therapy. Regardless of the method of reconstruction, the goals of surgery remain the same; these include creating a functional and esthetic phallus. We present a case of 49-year-old male diabetic presented for penile reconstruction after iatrogenic skin shaft sub-amputation post penile implant surgery complications done 1 year ago in a country where technical experts for this surgery are absent. Dartos Fascio-Myo-Cutaneous Flaps for penile skin loss is used with satisfactory results.

## INTRODUCTION

The ultimate goal of reconstructive penile surgery is to have a penis with normal function and appearance. The management of penile injury requires a wide variety of surgical techniques. Generally, the extent of the defect dictates the means of reconstruction. A surgical defect may range from one involving a single tissue or structure to a total penectomy defect, requiring microsurgical reconstructions. Scrotal flap used in the treatment of different kinds of penile defects has been described for reconstruction of patch defects of the penis [1, 2].

## CASE REPORT

**Figure 1:**
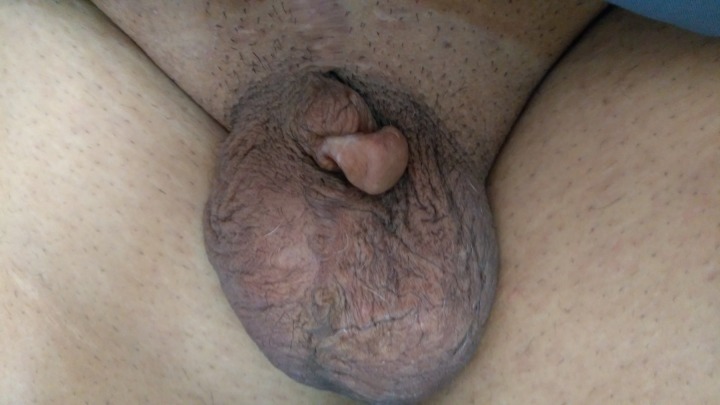
Preoperative image showed a small penile length &small glans tissue, length in flaccidity of 2 cm.

A 49-year-old male heavy smoker known to have diabetes type 2 since a few years ago, erectile dysfunction s/p complicated penile prosthesis surgery presented with penile skin shaft sub-amputation, history goes back to a few years ago when the patient reported erectile dysfunction for 3 years when he tried medical treatment with PDE5 inhibitor with a poor response, he decided to undergo penile implant surgery 1 year ago, the surgery was complicated and penile shaft sub-amputation was done. Examination of the genitalia revealed small penile length with small glans tissue, present in flaccidity of 2 cm (Fig. 1).

The consistency of the penis lacked elasticity, similar to a scar; most of the penis length was constricted inside the pubis, with normal scrotum and testicle. We elected to use the technique of scrotal dartos-fascio-myo-cutaneous flaps for penis elongation with few modifications.

**Figure 2:**
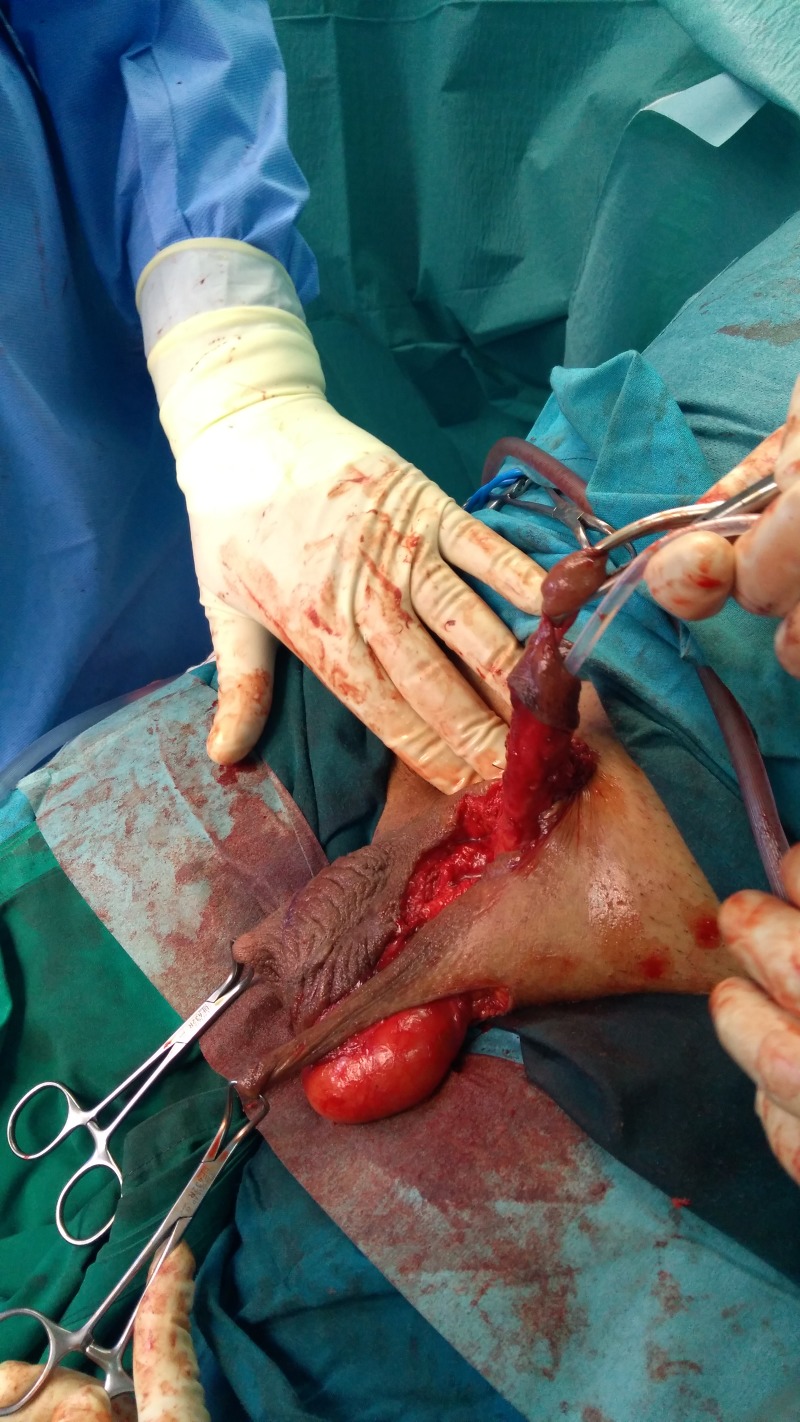
Intraoperatively: a two dartos-fascio-myo-cutaneous flaps were designed to cover the shaft.

At first, the adhesion scar tissue that entrapped the penis inside the pubis was released. When the shaft is mobilized, two dartos-fascio-myo-cutaneous flaps were designed to cover the shaft (Fig. 2). The flap pedicle was 5 cm in width and 8 cm in length while the scrotal raphe is considered as the longitudinal axis of the flap since the medial scrotal arteries ascending on each side are always incorporated. At the perineal scrotal junction, the incision lines separate toward the ischiatic tuberosities.

The flaps were rotated to cover the dorsal and ventral part of the shaft, the edges of neo-shaft were closed in an interrupted manner using tension-free suture then the scrotal defect was closed primarily.

The preoperative length in flaccidity was approximately 2 cm and the postoperative one was about 9 cm.

## RESULTS

Primary healing occurred in 14 days. No infection or flap necrosis was noted. Satisfied aesthetical appearance was obtained.

**Figure 3:**
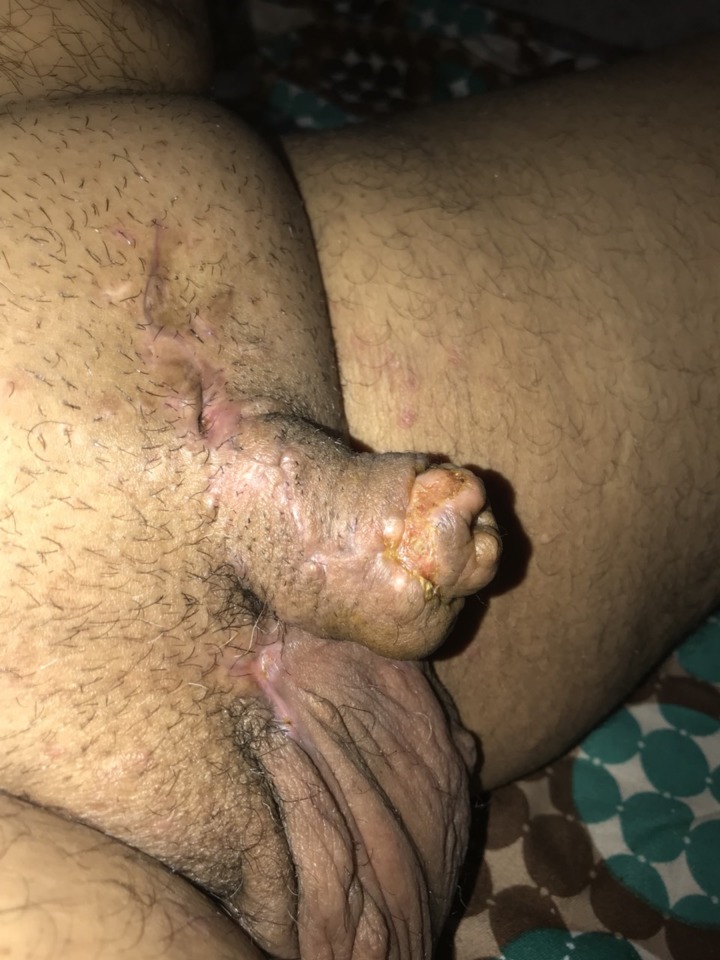
Penile form on post operative day 30.

The penile form on post operative day 30 (Fig. 3).

## DISCUSSION

Penile skin loss may occur as a consequence of trauma, carcinoma, infection, burns or iatrogenic causes such as excessive surgical excision during circumcision [3]. A skin graft is commonly used to resurface the denuded areas after skin necrosis. However, this simple and rapid approach has some inherent disadvantages, including paresthesia, contracture, and mismatched skin color. Several techniques are available for recovery of the length of the shaft, such as skin grafts, local or free flaps.

While pedicle flaps such as from groin skin or abdominal skin, rectus abdominis and gracilis have been used historically and recently for penile reconstruction, these lead to suboptimal results with poor esthetic and functional outcomes. Hence, microsurgical free flap reconstruction has become the method of choice for penile reconstruction. The ideal flap should be one that is sensate and hairless, with sufficient tissue to allow tubularization, as well as with a long pedicle. The radial forearm flap fulfills these requirements and is by far the most commonly used free flap for penile reconstruction [1].

The dartos- musculocutaneous flap is very suitable for coverage of the penile skin defect as the scrotal tissue is very elastic, not bulky and capable of great distension. Satisfactory reconstruction requires regaining a good aesthetical appearance, a valid recovery of functionality with redundant and durable skin envelope for complete erection providing acceptable sexual intercourse and satisfactory sensation.

This technique provides a good cosmetic appearance, functional outcomes, and excellent postoperative satisfaction grades [4].

The skin of the scrotum is the most similar to the skin of the shaft both for its color, thickness, elasticity, and consistency. The scrotum has a very high density of hair and it may require laser removal when used in the penile reconstruction. Fernandez et al, had dissected scrotal skin in fresh specimens in order to examine the scrotal circulation by transillumination test. They found an extremely rich vascular in both the dartos muscle and skin which made it safe to be raised as a musculocutaneous flap regardless of the pedicle location [5]. Jindarak et al used the bilateral scrotal flap technique to resurface the penile shaft and they noted that this technique can restore both the anatomy of the penile shaft and its function nearly to their normal status [6].
